# Neurobiology of Parental Regulation of the Infant and Its Disruption by Trauma Within Attachment

**DOI:** 10.3389/fnbeh.2022.806323

**Published:** 2022-04-07

**Authors:** Nina Graf, Roseanna M. Zanca, Wei Song, Elizabeth Zeldin, Roshni Raj, Regina M. Sullivan

**Affiliations:** ^1^Psychology Department and Center for Neural Science, New York University, New York, NY, United States; ^2^Emotional Brain Institute, The Nathan S. Kline Institute for Psychiatric Research, Orangeburg, NY, United States; ^3^Child and Adolescent Psychiatry, New York University Langone Medical Center, New York, NY, United States

**Keywords:** caregiver regulation, trauma bonding, attachment, homeostasis, social buffering, stress, mother, mother-infant dyad

## Abstract

The complex process of regulating physiological functions and homeostasis during external and internal disruptions develops slowly in altricial species, with parental care functioning as a co-regulator of infant physiological and emotional homeostasis. Here, we review our current understanding of the infant’s use of parental behaviors for neurobehavioral regulation and its disruption with harsh parental care. Taking a cross-species view, we briefly review the human developmental literature that highlights the importance of the caregiver in scaffolding the child’s physiological and emotional regulation, especially under threat and stress. We then use emerging corresponding animal literature within the phylogenetically preserved attachment system to help define neural systems supporting caregiver regulation and its supporting causal mechanism to provide translational bridges to inform causation and mechanisms impossible to define in children. Next, we briefly review animal research highlighting the impact of specific sensory stimuli imbedded in parental care as important for infant physiological and emotion regulation. We then highlight the importance of parental sensory stimuli gaining hedonic value to go beyond simple sensory stimuli to further impact neurobehavioral regulation, with poor quality of care compromising the infant’s ability to use these cues for regulation. Clinically, parental regulation of the infant is correlated with later-life neurobehavioral outcome and quality of life. We suggest an understanding of this parental regulation of the infant’s immediate neurobehavioral functioning within the context of attachment quality, that may provide insights into the complex processes during early life, initiating the pathway to pathology.

## Introduction

Homeostasis is a complex self-regulating process of maintaining physiological functions at optimal levels and engaging mechanisms to readjust with external and internal challenges (Cannon, 1929). Specifically, in response to internal and external challenges, the body self-regulates its physiological functioning to adjust myriad bodily processes, some of which involve emotional regulation. Homeostasis and self-regulation in response to challenges develop slowly in altricial mammalian species, such as humans, non-human primates, and rats. Sensory stimuli received during parental care are important for the infant to maintain homeostasis across myriad physiological and emotional systems until self-regulation is achieved (Kopp, 1982; Shields et al., 1994; Gunnar and Donzella, 2002; Bridgett et al., 2015; Montroy et al., 2016; Silvers et al., 2017; Li-Grining et al., 2019; Vink et al., 2020; Fotopoulou et al., 2021). The purpose of this review is to present our current understanding of the neurobehavioral response of the infant to parental caregiving and its importance to the infant’s short-term physiological and emotional regulation. We focus on the impact of parental care on infant neurobehavioral functioning, with some emphasis on one specific parental behavior–regulation of infant homeostasis. Next, we review the literature on infant adverse experiences and the disruption of parental regulation, to define an atypical developmental experience that goes beyond the initial adversity.

### Self-Regulation Is Immature in Infants of Altricial Species and Parental Care Provides the Sensory Stimulation Regulating Infant Neurobehavioral Function

Children are born with self-regulatory mechanisms in many physiological systems which are necessary for homeostasis and to support myriad processes to sustain life. Other physiological systems use parental co-regulation over the first months to years of life, of varying degrees.

For instance, food intake is heavily dependent on parental regulation, while body temperature can be regulated by the infant but the caregiver optimizes temperature through clothes and the warmth of physical contact (Fotopoulou et al., 2021). Frequently, the impact of maternal care on the infant and the degree of parental co-regulation is subtle, with its impact becoming visible with removal or dramatic changes in parental care. For example, young children can maintain homeostasis of vital functions, such as heart rate and respiration, but the regulatory role of parental care was shown through experiments comparing infants alone v. engaged in parental contact *via* somatosensory (temperature, touch), olfactory (caregiver odor), visual (face), and auditory (voice) stimulation (Kommers et al., 2019; Suga et al., 2019; Buhler-Wassmann and Hibel, 2021; Ionio et al., 2021). This regulation is also seen in the infant’s co-sleep with the parent, producing improved sleep compared to when the infants sleep alone (Mosko et al., 1997; Richard and Mosko, 2004; Waynforth, 2020; Yoshida and Funato, 2021).

Of course, physiological homeostasis includes neural mechanisms regulating emotional homeostasis. The child’s emotional regulation is enhanced by parents as evidenced by the more effective soothing of a crying child by the parent compared to a stranger engaging in similar comforting behaviors (Bridges et al., 1997; Feinberg et al., 2009; Hazen et al., 2010; Yoshida and Funato, 2021). In children, regular disruption of emotional homeostasis during early life (i.e., through adverse rearings such as deprivation or maltreatment) is highly correlated with later-life compromised functioning (i.e., psychiatric disorders, impaired academics, etc.), although specific causal mechanisms embedded within the infant-caregiver relationship have remained elusive (Raineki et al., 2012; Buhler-Wassmann and Hibel, 2021). The lack of understanding specific infant self-regulatory and co-regulatory mechanisms, such as specific parental behavior or sensory stimuli necessary and causal for infant physiological and emotional homeostasis, has hampered our understanding of the correlational link between infant dysregulation and later-life compromised outcome (Tronick et al., 1977; Feldman et al., 2002; Raposo et al., 2014; Cevasco-Trotter et al., 2019; Palacios-Barrios and Hanson, 2019; Tottenham, 2020; Vink et al., 2020). For example, it hasn’t been determined (1) how exactly an infant regulates its sleep-wake cycle, (2) if sleep-wake cycle dysregulation is a marker for behavioral or emotional difficulties, (3) how much it is self-regulated by the infant or supported by co-regulation from the parent, and (4) what specific parental behavior regulates or dysregulates the sleep-wake cycle of an infant (Scher, 2005; Whittingham and Douglas, 2014).

### Early Animal Models: Regulation by Maternal Care With Replacement of Sensory Stimuli

In the 1980s, Myron Hofer and others observed that children separated from the mother exhibited dysregulation of myriad physiological functions and behaviors (Hofer, 1981, 1994). Using rodent infant-mother dyads, Hofer operationalized the role of the parent in regulating infant physiology and behavior. Specifically, Hofer removed maternal care and questioned which sensory stimuli replacement would regain homeostasis. He uncovered specific causal mechanisms hidden in complex maternal behaviors that controlled very specific physiological functions of the infant rat, which he termed “hidden regulators.” For example, the mothers’ tactile stimulation regulates pups’ growth hormones, while the mothers’ phasic provision of milk regulates the pup’s sleep-wake states (Hofer, 1994, 2006). Of course, others also significantly contributed to this research. For example, removal of the mother could be partially reversed by artificial feeding and tactile stimulation (mimic licking) and repaired heightened adrenocorticotrophic hormone and growth hormone levels (Harlow, 1959; Powell et al., 1967; Schanberg et al., 1984; Schanberg and Kuhn, 1985; Pauk et al., 1986; Francis et al., 1999; Oers and Kloet, 1999). Overall, this research went beyond the global “maternal behavior” as causal, to specific links between subcomponents of maternal behavior and homeostasis of specific systems. This was a paradigm shift in our understanding of the importance of maternal care that provided the foundation for a new approach to the care of young infants (Schanberg and Field, 1987; Reite et al., 1989; Field, 2003; Brett et al., 2015; Porges et al., 2019; Fotopoulou et al., 2021; Yoshida and Funato, 2021).

### Regulation With the Caregiver Exceeds That Achieved by Sensory Stimulation Alone

**FIGURE 1:**
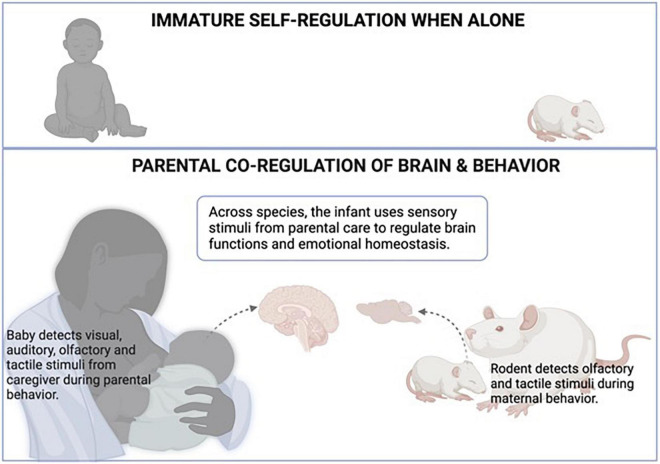
Attachment to the primary caregivers is learned across species. Once the infant learns the attachment figure, the attachment figure acquires special value for the infant, including regulation of homeostasis (Bowlby, 1969; Hofer, 1994). As illustrated in the top panel, when alone, the infant’s ability to maintain physiological and emotional homeostasis is limited. As illustrated in the lower panel, the regulatory role of sensory stimuli from the attachment figure was shown to have a particular impact on the infant’s brain to permit homeostasis and regulation of diverse systems. During maternal care, the infant detects visual, auditory, olfactory, and tactile stimuli from the caregiver during parental behavior, while rodents rely on olfactory and tactile stimuli during maternal behavior. Across species, the infant uses these sensory stimuli from parental care to regulate brain functions, which extends to physiological and emotional homeostasis. Created with BioRender.com.

More recently, research is suggesting that sensory stimuli infants experience during interactions with the caregiver acquire hedonic value that provides potent capabilities to regulate the infant more robustly (Perry et al., 2016). Indeed, work by Myron Hofer’s team and others hinted that some features of the mother rat could not be duplicated by simple sensory stimulation: for example, the rat pup’s behaviors could not be altered by a neutral novel odor and required the presentation of the mother’s odor (Hofer, 1994). This is supported by observations of a young child interacting (or being hugged) with the parent as opposed to a stranger, which illustrates the infant’s specialized prosocial behaviors to the biological/adoptive (learned) caregiver compared to strangers (Singer et al., 1985; Altenhofen et al., 2013; Tottenham et al., 2019; Yoshida et al., 2020). Overall, this research suggests that young children are using all of their sensory systems to use parental information to maintain homeostasis, as illustrated in Figure 1.

Also illustrated in Figure 1, other animal species, such as rodents, also use maternal behavior and sensory cues to maintain homeostasis and provide a translational bridge and opportunities to explore neural mechanisms using invasive techniques unsuitable to use on children. For example, rodents can provide a temporally dynamic view of the infant’s brain during mother-infant interactions. Using local field potentials (LFP), we recorded the prefrontal cortex (PFC) of very young pups, targeting a brain region known to be responsive to alteration in maternal care (Opendak et al., 2020). These recordings of neural oscillations illustrated the profound and immediate impact of the maternal presence and maternal behavior on pups’ neural oscillations. For example, when pups are near (not touching) the mother compared to alone, pups’ LFP shows a reduction in high-frequency bands. Contact with the mother produces a further reduction, and maternal behaviors produce transient, rapid increases in LFP power: nipple attachment increased slow-wave activity, while maternal stimulation such as grooming and milk ejection produced very rapid and transient cortical desynchronization (Opendak et al., 2020). The neural controls of these PFC oscillations are complex: Courtiol et al. (2018) have shown that the mother’s presence regulates the activity of cortical oscillations by increasing low-frequency oscillations mediated by the serotonin receptor, 5-hydroxytryptamine-2-receptor, while the transient cortical desynchronization induced by grooming and milk ejection is dependent on norepinephrine (Sarro et al., 2014). Overall, this data illustrated the robust and temporally dynamic impact on pups’ brain rhythmic oscillations, thus impacting a process shown to be critical in guiding brain development (Penn and Shatz, 1999).

### Using Parental Reduction of Infant Fear for Understanding Emotional Regulation

Parental reduction of their child’s fear was noted in Bowlby’s Attachment Theory in the 1960s: children in novel or threatening environments showed less fear if their parent was present (Bowlby, 1965, 1969). Expanding on this observation, more recent research showed that the impact of maternal presence on children is particularly salient during the regulation of the threat response: more specifically, maternal presence reduces the child’s fear and is accomplished through suppression of both stress hormone and amygdala activity, as indicated by correlations (Gunnar and Donzella, 2002; Gee et al., 2014; Hostinar et al., 2015; Callaghan et al., 2019). Animal studies showed that this effect is also seen in diverse altricial animal species (Suchecki et al., 1993; Wiedenmayer et al., 2003; Shionoya et al., 2007; Hennessy et al., 2009; Sarro et al., 2014; Sanchez et al., 2015; Sullivan, 2017; Sullivan and Opendak, 2021), which permitted the identification of a causal link between infant fear and maternal presence. Indeed, we now understand maternal presence suppressing infant fear because her presence suppresses the basolateral subarea of the amygdala, a brain area well-documented to support fear in pups and adults (Moriceau et al., 2006; Moriceau and Sullivan, 2006; Shionoya et al., 2007). How the infant detects the mother to then suppress the amygdala is also known: the smell of the mother’s odor enters the pup’s olfactory system and travels to the hypothalamic paraventricular nucleus to suppress activation of the hypothalamic-pituitary-adrenal (Shionoya et al., 2007). The infant amygdala has an age-specific dependence on the stress hormone corticosterone (CORT), and its suppression by the mother is sufficient to block mechanisms (Thompson et al., 2008). Thus, maternal suppression of CORT deprives the infant amygdala of the plasticity mechanisms required for its activation. This plasticity suppression also means the infant amygdala cannot support amygdala-dependent fear learning (Moriceau et al., 2006; Moriceau and Sullivan, 2006; Shionoya et al., 2007), which was recently replicated in children (Tottenham et al., 2019) along with suppression of the amygdala (Gee et al., 2014).

Further capitalizing on our ability to probe the rat brain during interactions with the mother, we focused on the role of the neurotransmitter dopamine (DA) because it’s documented to be altered by maternal care and presence in infant rats (Tamborski et al., 1990; Andersen et al., 1992; Barr et al., 2009; Opendak et al., 2021). We began by using microdialysis, a technique that enables us to measure DA levels in pups during maternal presence, to show that maternal presence blocks DA release into the basolateral amygdala (Barr et al., 2009). Using brain dissection, we showed that the mother also blocked AMPA receptors and plasticity molecules (Opendak et al., 2019) showing the mother was altering the pups global amygdala activity, its connectivity to other brain regions, but also the intracellular machinery within amygdala neurons controlling pups ability to use brain areas to alter behavior and learning (Moriceau and Sullivan, 2006; Barr et al., 2009; Opendak et al., 2019; Robinson-Drummer et al., 2019; Sullivan and Opendak, 2021). For reviews see Kikusui et al. (2006), Gunnar et al. (2015), Sanchez et al. (2015), Al Aïn et al. (2017), Kiyokawa and Hennessy (2018).

While a present and calm parent typically suppress their offspring’s fear, an agitated or fearful parent can enhance their offspring’s fear *via* social transmission (Chang and Debiec, 2016; Silvers et al., 2021). Rodent and human research have shown that a parent expressing fear to a specific cue will transmit that specific fear to their child, a process associated with heightened amygdala activation. Using rodents to identify causal mechanisms, a learned fearful odor presented to the mother is sufficient to induce an amygdala-dependent, learned odor-specific fear in pups, if the mother had expressed fear in the pups’ presence (Debiec and Sullivan, 2014; Rickenbacher et al., 2017). Specifically, rat mothers were fear-conditioned (peppermint-shock pairing either before mating or during pregnancy) and presented with the odor conditioned stimulus (CS) to express fear in the presence of their pups. Pups immediately showed fear of the peppermint odor and continued to show amygdala-dependent fear of the peppermint odor the next day, whether or not the mother was present. The mechanism for this social transmission is socially communicated: maternal fear expression is accompanied by the release of a fear pheromone (potentially comparable to the child seeing a fearful parental facial expression), which increases pups CORT and amygdala activity to support fear learning (Debiec and Sullivan, 2014; Boulanger-Bertolus et al., 2017). This social transmission of fear occurs throughout the lifespan (Askew and Field, 2008; Chang and Debiec, 2016).

### Disrupting Parental Regulation Through Early Life Adversity

To acknowledge how effective co-regulation works, it’s important to understand what happens when parental regulation is disrupted and its effectiveness decreased. For example, even an abused child learns and expresses attachment to their caregiver, which has also been seen in other animal species (Ainsworth, 1969; Perry and Sullivan, 2014), although self-regulation appears compromised with trauma-related attachment (Shields et al., 1994). This abuse-related attachment occurs across species: maltreated chicks following the imprinting figure (Hess, 1962; Salzen, 1970; Rajecki et al., 1978), infant dogs shocked by a human caregiver seek that person (Stanley and Elliot, 1962) and infant monkeys inflicted with pain by a wire surrogate or an abusive mother continue to show attachment (Harlow and Harlow, 1965; Maestripieri et al., 1999; Sanchez et al., 2001; Suomi, 2003; O’Connor and Cameron, 2006). Animal research on rodents suggests the evolutionarily conserved attachment system appears to rely on an attachment learning circuit that is equally responsive to rough and nurturing maternal care to support attachment learning (Sullivan et al., 2000; Sullivan, 2012).

While there are short-term benefits to constructing an attachment system that ensures an infant learns to attach regardless of the quality of care, this system does produce robust detrimental long-term outcomes that primarily emerge around peri-adolescence. Finding biomarkers to early life that can be used to predict later life problems is a major focus of the adversity developmental research. However, subtle immediate behavioral identification of children who have compromised attachment has been shown experimentally by Ainsworth and the stressful Strange Situation Procedure (SSP, i.e., repeated parent-child separations and reunions) (Ainsworth, 1969). Specifically, Ainsworth’s SSP showed that parents can typically calm (regulate emotions) the stressed child, although maltreated children instead show ambivalent, contradictory, and incomplete/undirected behavioral responses, termed Disordered Attachment. Disordered attachment is the only attachment category associated with later life pathology (Main and Solomon, 1990; Mason et al., 2005).

To better understand the role of maternal regulation (or lack of) in disrupted SSP social behavior toward the mother, we developed an SSP test in rodents. This also builds on our previous research showing that pups reared by a maltreating mother have a significantly slower approach response to the mother outside the nest and a blunted neural response to the mother across brain areas that include the amygdala, PFC, and hippocampus (Rincon-Cortes and Sullivan, 2016; Al Aïn et al., 2017; Yan et al., 2017; Perry et al., 2019). We replicated these results in the rodent SSP during the final infant-mother reunion and extended these results to show that the typical dynamic cortical LFP response pups show to the maternal presence and maternal behaviors were significantly blunted. Capitalizing on the power of animal models to define mechanisms, we systemically pharmacologically block pups’ stress hormone release during the SSP, which repaired pups’ disrupted behavior to the mother, and pups regained a significant amount of dynamic LFP in response to the mother (Opendak et al., 2020). The mother was anesthetized (no maternal behaviors) in our SSP test, which was required to mimic the human SSP.

However, to better understand pups’ response to maternal behaviors, we also tested pups during natural interactions with the mother as we recorded cortical LFP. Maltreated pups’ response to maternal care was also blunted, but only to nurturing maternal care. Specifically, the LFP response to harsh maternal care (low occurrence in control pups permits comparison), such as stepping on or dragging pups did not differ between control and maltreated pups, while the LFP response to the maternal nurturing care (i.e., nipple attachment, grooming) was blunted. Importantly, while these LFP measures uncovered differences in pups’ neural response within the nest, even when no behavioral differences were found within the nest–the stress of the SSP was required to uncover pups’ behavioral differences (Opendak et al., 2020).

**FIGURE 2:**
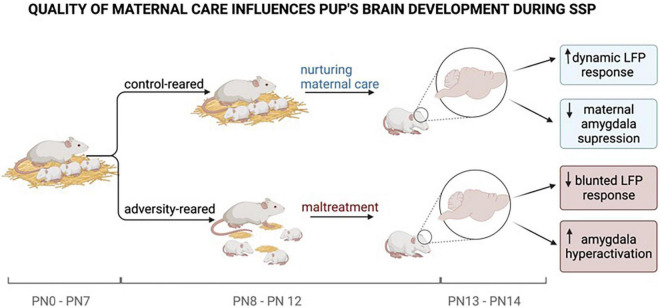
Experiments by Opendak et al. (2020) have shown that pups reared under adverse conditions face repeated maltreatment by their mothers compared to control-reared pups. The quality of maternal care provided is causal for the pup’s brain development. While typically reared pups show a dynamic LFP response (synchronized, low-frequency waves) and a maternally suppressed amygdala response, adversity reared pups to show a blunted LFP response (desynchronized, high-frequency waves) and amygdala hyperactivation. At PN 10–12 the pup’s brain is most sensitive to maternal regulation. The mother’s ability to regulate the pup’s physiological and emotional state diminishes as the pup is getting older and more independent. Neurological changes during brain development caused by maternal maltreatment set the early stage for later-life pathologies. Created with BioRender.com.

As illustrated in Figure 2, our integration of these surprising results illustrates that disfunction of maternal regulation of the infant is a sensitive measure of early life pathology and appears to be a robust measure in situations ranging from threat (fear learning) and a stressful novel situation (Ainsworth’s SSP) where both behavior and brain showed atypical responses. Within the nest, where behavioral pathologies are difficult to locate, the LFP response to the mother was atypical and similar to the blunted response found in stressful testing. Thus, infant behavioral effects of maltreatment are subtle, although its disruption of maternal regulation of the infant appears consistent across myriad situations.

## Conclusion

Sensory stimulation in early life has been considered important since the 1950s and further refined in the 1980s by Hofer as sensory stimulation as “Hidden Regulators” of pup physiology. More recently, we have begun to understand that sensory stimuli received from the parent are more than simple sensory stimuli–they’re sensory stimuli that have acquired special value through their learned association with the attachment figures and have significantly more robust strength to alter the infant’s neurobehavioral function. The SSP animal model approach aligns with decades of research on children, highlighting the parent’s special role in guiding the infant’s interaction with the world and as a source of comfort. This review of animal models of human regulation has highlighted that maternal presence regulates the brain on myriad levels, including gene expression, receptors, neurotransmitters, brain regions of interest, circuits, and networks across the brain including data from our lab. This regulatory effect occurs during parent-infant interactions but is more salient during a threat. Further research focusing on understanding what exactly has gone wrong within a parent-infant interaction as well as its neural underpinnings can help in targeted interventions and treatments to repair the relationship and prevent later-life pathologies.

## Author Contributions

NG and RMS wrote the manuscript. RMZ contributed specific paragraphs to the manuscript. WS and RR made the figures on BioRender.com. EZ reviewed the manuscript for orthographic mistakes. All authors read and approved the manuscript.

## Conflict of Interest

The authors declare that the research was conducted in the absence of any commercial or financial relationships that could be construed as a potential conflict of interest.

## Publisher’s Note

All claims expressed in this article are solely those of the authors and do not necessarily represent those of their affiliated organizations, or those of the publisher, the editors and the reviewers. Any product that may be evaluated in this article, or claim that may be made by its manufacturer, is not guaranteed or endorsed by the publisher.
